# Improved physiological and morphological traits of root synergistically enhanced salinity tolerance in rice under appropriate nitrogen application rate

**DOI:** 10.3389/fpls.2022.982637

**Published:** 2022-07-29

**Authors:** Yinglong Chen, Yang Liu, Jianfei Ge, Rongkai Li, Rui Zhang, Yang Zhang, Zhongyang Huo, Ke Xu, Huanhe Wei, Qigen Dai

**Affiliations:** ^1^Key Laboratory of Saline-Alkali Soil Improvement and Utilization (Coastal Saline-Alkali Lands), Jiangsu Key Laboratory of Crop Genetics and Physiology, Jiangsu Key Laboratory of Crop Cultivation and Physiology, Ministry of Agriculture and Rural Affairs, Jiangsu Co-innovation Center for Modern Production Technology of Grain Crops, Research Institute of Rice Industrial Engineering Technology, Yangzhou University, Yangzhou, China; ^2^College of Environmental Science and Engineering, Yangzhou University, Yangzhou, China

**Keywords:** rice (*Oryza sativa* L.), salt stress, yield, root, nitrogen

## Abstract

Numerous papers studied the relations between nitrogen rate and rice yield in saline soils, whereas the rice root morphological and physiological characteristics mediating nitrogen rates in yield formation under varied salinity levels remain less concerns. Through a field experiment applied with five nitrogen rates (0, 210, 255, 300, 345, and 390 kg ha^–1^) in saline land, we found that rice yield peaked at 7.7 t ha^–1^ under 300 kg ha^–1^ nitrogen, and excessive N was not conductive for increasing yield. To further elucidate its internal physiological mechanism, a pot experiment was designed with three N rates (210 [N1], 300 [N2], 390 [N3] kg ha^–1^) and three salt concentrations (0 [S0], 1.5 [S1], 3.0 [S2] g kg^–1^ NaCl). Results showed that the average grain yield was decreased by 19.1 and 51.1% under S1 and S2, respectively, while notably increased by 18.5 and 14.5% under N2 and N3, respectively. Salinity stress significantly inhibited root biomass, root length and surface area, root oxidation capacity (ROC), K^+^ and K^+^/Na^+^ ratio, and nitrogen metabolism-related enzyme activities, whereas root Na^+^ and antioxidant enzyme activities were notably increased. The mechanism of how insufficient N supply (N1) affected rice yield formation was consistent at different salinity levels, which displayed adverse impacts on root morphological and physiological traits, thereby significantly inhibiting leaf photosynthesis and grain yield of rice. However, the mechanism thorough which excessive N (N3) affected yield formation was quite different under varied salinity levels. Under lower salinity (S0 and S1), no significant differences on root morphological traits and grain yield were observed except the significantly decline in activities of NR and GS between N3 and N2 treatments. Under higher salinity level (S2), the decreased ROC, K^+^/Na^+^ ratio due to increased Na^+^, antioxidant enzyme activities, and NR and GS activities were the main reason leading to undesirable root morphological traits and leaf photosynthesis, which further triggered decreased grain yield under N3 treatment, compared to that under N2 treatment. Overall, our results suggest that improved physiological and morphological traits of root synergistically enhanced salinity tolerance in rice under appropriate nitrogen application rate.

## Introduction

There is about 1,125 million hectares saline land in the world (Hossain, 2019). As an important reserve cultivated land resource, saline-alkali land is without doubt vital for the increasing global demand for food, due to the rapidly growing population which is predicted to reach approximately 9 billion by 2050 (van der Mensbrugghe et al., 2009). In China, saline-alkali soils account for 25% of farmland and are underutilized (Zhang et al., 2022). However, most edible crops are prone to soil salinity, with notable decreased production and productivity, making it a challenge for human to well utilize saline-alkali land (Stille et al., 2011).

Rice (*Oryza sativ*a L.) is one of the major staple crop species, feeding more than half of the world. Compared with wheat, barley and other cereal crops, rice displays more sensitive to salinity (Munns et al., 2006). With the advantage of irrigated aquatic, a great portion of rice in China is grown along the coastal areas suffering various degrees of salinity, where rainfall and river-water resources are relatively abundant (Chen et al., 2021).

Numerous research papers have focused on salinity and its adverse effects on crops. Salt stress drastically decreases the rice tillering, shoot dry weight, and final grain yield. Soil salinization causes soil nutrient loss, destroys the structure of soil aggregates, and significantly limits crop growth and yield formation (Kordrostami et al., 2017). Salinity causes intracellular osmotic stress and ion toxicity (mainly Na^+^ toxicity), thereby inducing insufficient absorption of nutrients such as nitrogen, phosphorus, potassium, and calcium, and triggering many aspects of shielding responses such as morphological and physiological changes, as well as related molecular events (Wang et al., 2013; Al-Tamimi et al., 2021; Islam et al., 2021). Enhanced antioxidant enzyme activities and disrupted K^+/^Na^+^ homeostasis are frequently mentioned where salinity stress is involved in rice (Hussain et al., 2018).

Root is the principal organ directly affected by salinity stress as it is exposed to salts in the rhizosphere (Munns and Sharp, 1993). Root is not only the main absorption organ for plants to obtain water and nutrients from the soil, but also the organ for the synthesis, assimilation and transformation of life substances such as amino acids and hormones in the plant (Seo et al., 2020). Therefore, the normal growth and development of the root system is very important to plant growth. The morphological and physiological characteristics of root system under salt stress are essential indicators that directly reflect the salinity tolerance of rice (Jharna et al., 2017). It’s reported that there was a significant positive correlation between root biomass and crop yield under salt stress (Munns and Tester, 2008; Annunziata et al., 2016). A higher root biomass, root length density, and root oxidation activity contributed to higher grain yield formation (Chu et al., 2014).

Nitrogen is an important nutrient element for rice growth and yield formation. Appropriate nitrogen application contributes to high rice yield, while excessive nitrogen application may have adverse effects, especially in saline land (Murtaza et al., 2000). Reasonable nitrogen application can promote crop root growth and root vigor, improve water and fertilizer absorption capacity, and enhance plant resistance to adverse conditions. It’s reported that increasing nitrogen fertilizer application in saline land can increase the absorption and upward transportation of K^+^ in plants, and reduce the Na^+^ content of shoots, thereby facilitating the accumulation of dry matter (Ahanger et al., 2019; Singh et al., 2019).

Most of the previous literatures have studied the nitrogen rate on rice yield and grain quality formation (Seemann and Sharkey, 1986; Rios-Gonzalez et al., 2002; Choi et al., 2004). However, the rice root morphological and physiological characteristics in response to varied nitrogen rates and salinity levels remains to be least researched area. It is with this backdrop we hypothesized that (a) whether N supplementation modulates antioxidant, K^+/^Na^+^ homeostasis and nitrogen metabolism for enhanced rice salinity tolerance, and (b) whether the mechanisms varied in affecting yield formation between insufficient and excessive nitrogen application rates.

## Materials and methods

### Experiment materials and experimental designs

#### Field experiment

**FIGURE 1:**
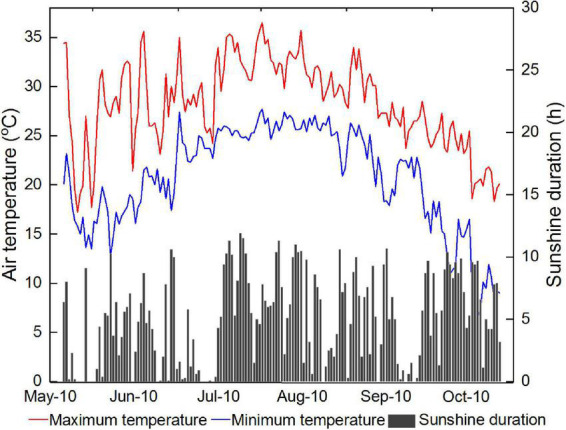
The daily maximum temperature, daily minimum temperature and daily sunshine duration during rice sowing and full-ripening period in the year of 2018.

The field experiment was carried out at Jinhaidao experimental base of Yangzhou University, Jiangsu Province, China (33^°^57′ N, 120^°^ 24′ E) in 2018. The daily maximum temperature, daily minimum temperature, daily mean temperature, total sunshine duration and total rainfall were 28.3°, 20.7°, 24.1°C, 799 h and 698 mm, respectively, during rice sowing date and full-ripening period (Figure 1). The soil in the experimental field has a sandy loam texture with 0.46 g kg^–1^ total nitrogen (N), 20.5 mg kg^–1^ Olsen phosphorus (P), 85.6 mg kg^–1^ available potassium (K) and 2.7 g kg^–1^ organic carbon in the 0–20 cm soil layer. The soil conductivity was 5.5–6.2 mS cm^–1^ and the pH was 8.0–8.5. Rice cultivar Nanjing 9108 was adopted and five nitrogen rates (0, 210, 255, 300, 345, and 390 kg ha^–1^) were arranged in the experiment. Nitrogen fertilizer was split-applied as follows: 30% basal, 15% at 7 days after transplanting, 40% at the panicle initiation stage, and 15% at the booting stage. Moreover, 600 kg ha^–1^ calcium superphosphate was applied as basal fertilizers before transplanting for all treatments. Rice seeds were sowed on May 15, rice seedlings were transplanted on June 19 and harvested on October 20.

#### Pot experiment

The pot experiment was conducted at the experimental base of Yangzhou University, Jiangsu Province, China (32^°^ 30′ N, 119^°^ 25′ E) in 2019. The pots were arranged in the trial zone covered with a transparent waterproof top during the rice-growing season (May to October). Rice cultivars Nanjing 9108 was adopted in this experiment and synchronously arranged with three nitrogen rates (210 [N1], 300 [N2], 390 [N3] kg ha^–1^) and three salt concentrations (0 [S0], 1.5 [S1], 3.0 [S2] g kg^–1^ NaCl, the conductivity of the water layer was equivalent to 0, 3.15 and 5.82 mS cm^–1^, respectively). The conductivity of each treatment was monitored and controlled during the whole rice growing stage. The nitrogen fertilizer application method and phosphorus fertilizer application rate were consistent with the field experiment. The pot was 25 cm in diameter and 30 cm in height. Each pot was filled with 15 kg of sieved fine soil and planted with 16 seedlings evenly in 4 hills, and all treatments have twenty pots as replicates. Rice seeds were sowed on May 15, rice seedlings were transplanted on June 10 and harvested on October 22.

### Determination of rice yield and yield components

For the field experiment, two hundred representative hills of rice plants in each plot excluding border plants were harvested for determining grain yield at 14% moisture. In addition, one hundred representative hills of plants in each plot were cultivated for analyzing panicle traits and grain yield components. For the pot experiment, the grain yield and yield components were determined from 5 pots of each treatment at the full-ripening stage.

### Determination of root morphological traits and root oxidation capacity

At tillering, booting, heading, and full-ripening stages (noted as TR, BT, HD, and FR, respectively), rice plants were removed from 3 pots and rice roots were rinsed clean to determine root length and surface area through an WinRHIZO Root Analysis Software (Regent Instruments Inc., Quebec, Canada), followed by the determination of root dry weight. The remaining roots were used to measure root oxidation activity by measuring the oxidation of alpha-naphthylamine (α-NA) as described by Ramasamy et al. (1997). The root tip (0–2 cm) samples were collected and immersed in liquid nitrogen and then stored at −80°C for subsequent analysis.

### Determination of Na^+^ and K^+^ content

Dry samples (0.3 g) were crushed and digested with HNO_3_ and HClO_4_ (4:1, v/v), and then concentrated in a microwave oven (Mars, CEM Inc., New York, United States). The final K^+^ and Na^+^ concentrations were detected with atomic absorption spectrometry (PinAAcle 900, PerkinElmer Life and Analytical Sciences, Inc., Shelton, United States).

### Determination of antioxidant enzyme activities

Frozen root samples (0.3 g) were crushed in 5 mL of 0.1 M Tris–HCl buffer (pH 7.8) including 1 mM dithiothreitol, 1 mM EDTA and 1% polyvinyl pyrrolidone to obtain the homogenate, which was subsequently centrifuged at 20,000 ×*g* for 20 min at 4°C. The clear supernatant was obtained and used to measure enzyme activities. The superoxide dismutase (SOD; EC 1.15.1.1) activity was analyzed by calculating the nitro blue tetrazolium (NBT) photoreduction according to Jin et al. (2008). The peroxidase (POD; EC 1.11.1.7) activity was determined by analyzing the guaiacol oxidation at 470 nm according to Jiang et al. (2021) and the catalase (CAT; EC 1.11.1.6) activity was assayed as the absorbance decrease at 240 nm that accompanied H_2_O_2_ consumption following the method of Panda et al. (2017).

### Determination of leaf net photosynthesis rate

The leaf *P*n was monitored with a portable photosynthesis system (LI-6400, LI-COR, Lincoln, NE, United States) at 9:00–11:00 a.m. on a sunny day. The instrument parameters were adjusted according to Gu et al. (2017). The air temperature, PPFD, and CO_2_ concentration were controlled at 29–30°C, 1400 μmol m^–2^ s^–1^ and 380 μmol mol^–1^.

### Determination of nitrogen metabolism-related enzyme activities

Leaf samples were collected at heading stage. The activity of nitrate reductase (NR), glutamine synthetase (GS) and glutamate synthase (GOGAT) were determined with the specific assay kits (Comin Biotechnology Co., Ltd., Suzhou, China). Leaf samples were ground in a chilled mortar containing 6 ml of extraction buffer. NR activity was determined with a modified method reported by Savidov et al. (1997), GS activity was determined following the method of Han et al. (2017), and GOGAT activity was determined by spectrometer according to Liu et al. (2016).

### Statistical analysis

The data were subjected to analysis of variance with SPSS ver. 17.0. The least significant difference (LSD) test was performed to identify differences at significant level of 5 and 1%. All figures were designed with the Origin 8.0 software program.

## Results

### Rice grain yield and yield components

As indicated in Table 1, with the increase of nitrogen rate, the filled-kernel percentage and kernel weight displayed decreased trend, while the spikelets per panicle was significantly increased. The changing trends of panicles number and final rice grain yield were consistent under different nitrogen rates, both of which were peaked at 300 kg ha^–1^ nitrogen. The grain yield reached 7.7 t ha^–1^ under 300 kg ha^–1^ nitrogen, increased by 120% compared to that under 0 nitrogen.

**TABLE 1:** Effects of different nitrogen rates on rice yield and yield components in saline-alkaline field.

Nitrogen rate (kg ha^–1^)	Filled-kernel percentage (%)	Spikelets per panicle	Kernel weight (mg)	Panicles per m^2^	Grain yield (t ha^–1^)
0	88.7a	113.3d	23.7a	163.3d	3.5d
210	81.3b	121.9c	21.8b	304.4c	6.0c
255	80.5b	124.3bc	20.4c	342.2b	6.7bc
300	75.7c	127.6bc	20.5c	412.2a	7.7a
345	72.6c	131.1ab	20.2c	408.3a	7.0ab
390	71.1c	135.9a	19.4c	405.5a	6.8b

The values in the same column followed by different letters indicate statistical significance at the 0.05 probability level.

The filled-kernel percentage, spikelets number per panicle, kernel weight, rice panicle number, and final grain yield were significantly decreased under salinity stress compared with those of non-salt treatment, and the magnitude of reduction increased with increasing salt concentration (Table 2). The average grain yield was decreased by 19.1 and 51.1% under S1 and S2, respectively, compared with that under S0 treatment. Effects of nitrogen rate on grain yield and yield components in the pot experiment were consistent to those in the field experiment. Under different salt concentrations, rice grain yield under 300 kg ha^–1^ nitrogen showed higher than those under the other two nitrogen rates. A significant combined effect between salt and nitrogen occurred on spikelets number per panicle, panicle number and grain yield.

**TABLE 2:** Effects of different salt concentrations and nitrogen rates on rice yield and yield components.

Treatment	Filled-kernel percentage (%)	Spikelets per panicle	Kernel weight (mg)	Panicles per pot	Grain yield (g pot^–1^)
S0N1	96.0a	114.4b	24.1a	39.5b	104.5c
S0N2	95.4a	123.5a	24.0a	43.2a	123.3a
S0N3	95.2a	124.5a	23.8a	42.6a	120.2b
S1N1	93.7b	101.1c	23.6b	40.2d	87.4d
S1N2	94.2b	102.4c	24.1a	42.4b	98.6e
S1N3	93.5b	106.4c	23.4b	41.0c	95.4d
S2N1	82.7d	80.7e	21.3d	35.1f	48.2g
S2N2	87.8c	90.2d	22.1c	36.7e	62.6f
S2N3	86.0c	89.7d	20.8d	37.0e	59.3g
	**Average of salt treatment**				
S0	95.5a	120.8a	24.0a	41.8a	116.0a
S1	93.8b	103.3b	23.7b	41.2b	93.8b
S2	85.5c	86.9c	21.4c	36.3c	56.7c
	**Average of nitrogen treatment**				
N1	90.8b	98.7b	23.0a	38.3b	80.0c
N2	92.5a	105.4a	23.4a	40.8a	94.8a
N3	91.6a	106.9a	22.7b	40.2a	91.6b
		**Significance of factors**			
S	**	**	**	*	**
N	ns	*	*	*	**
S × N	ns	*	ns	**	**

S0, S1, and S2 indicate 0, 1.5, and 3.0 g kg^–1^ NaCl, respectively. N1, N2, and N3 indicate 210, 300, and 390 kg ha^–1^ nitrogen rate, respectively.

Values followed by a different letter within the same column are significantly different at P = 0.05 probability level; ns, not significant.

*, **: Significant at the P < 0.05 and 0.01 levels, respectively.

### Rice root morphological traits and root oxidation capacity

As shown in Table 3, rice root dry weight, root length and root surface area showed a unimodal curve which increased from tillering until it peaked at heading stage and then declined until full-ripening stage. All the three root morphological traits were significantly decreased under NaCl treatment, and the magnitude of reduction increased with increasing salt concentration. However, the effect of nitrogen on rice root morphological traits varied under different salt concentration. Under lower salinity (S0 and S1), increased nitrogen rate significantly improved root dry weight, length and surface area at all the four stages, whereas there was no significant difference on root length between N2 and N3 treatment. Under higher salinity (S2), the three root traits showed a unimodal curve with the increase of nitrogen rate after heading stage.

**TABLE 3:** Effects of different salt concentrations and nitrogen rates on rice root morphological traits.

Treatment	Root dry weight (g pot^–1^)				Root length (m pot^–1^)				Root surface area (cm^2^ pot^–1^)			
	TR	BT	HD	FR	TR	BT	HD	FR	TR	BT	HD	FR
S0N1	4.18a	7.03b	11.2c	8.82c	216.6a	380.4b	583.2b	489.5b	5621.7b	7625.7b	8215.7c	7215.5c
S0N2	4.26a	7.32a	12.45a	10.68a	218.0a	426.5a	630.3a	521.7a	5783.5a	8023.1a	9818.0a	9053.7a
S0N3	4.35a	7.25a	11.73b	9.54b	219.4a	435.4a	645.2a	518.7a	5864.2a	8267.5a	8925.7b	8215.7b
S1N1	2.84b	5.84d	8.95f	7.32d	163.4b	301.2d	501.2c	426.3c	4012.8d	5625.0d	6821.3f	5872.6f
S1N2	2.90b	6.25c	10.42d	9.46b	166.9b	356.0c	569.7b	496.7b	4534.7c	6028.2c	8607.8d	6928.4d
S1N3	2.96b	6.23c	9.84e	8.77c	166.2b	360.2c	572.4b	489.2b	4428.9c	6204.7c	7658.2e	6502.5e
S2N1	1.33c	4.20f	6.22i	5.03g	86.2d	174.2f	258.5f	204.3f	2729.6f	4218.6f	4922.7i	4463.2i
S2N2	1.42c	5.18e	7.02g	6.61e	92.4c	210.4e	325.7d	266.8d	3028.1e	5219.8e	6032.6g	5469.3g
S2N3	1.44c	5.24e	6.88h	5.69f	90.3c	198.6e	291.1e	252.4e	3000.5e	5005.6e	5301.5h	5008.0h

S0, S1, and S2 indicate 0, 1.5, and 3.0 g kg^–1^ NaCl, respectively. N1, N2, and N3 indicate 210, 300, and 390 kg ha^–1^ nitrogen rate, respectively. TR, BT, HD, and FR indicate tillering, booting, heading, and full-ripening stage, respectively.

The values in the same column followed by different letters indicate statistical significance at the 0.05 probability level.

**FIGURE 2:**
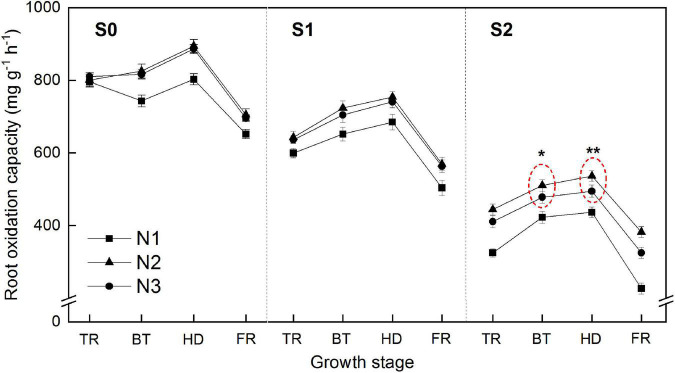
Effects of different salt concentrations and nitrogen rates on rice root oxidation capacity. S_0_, S_1_ and S_2_ denote salt concentrations of 0, 1.5, and 3.0 g kg^–1^ NaCl, respectively; N1, N2 and N3 denote nitrogen rates of 210, 300, and 390 kg ha^–1^. The data (*n* = 3) are mean values ±SD, calculated from three independent experiments. *, **: Significant at the *P* < 0.05 and 0.01 levels, respectively.

Root oxidation capacity (ROC) increased with rice growth stage and peaked at heading stage (Figure 2). Salinity had significant adverse effect on root ROC. The average ROC across all the four stages was decreased by 17.2 and 47.0% under S1 and S2, respectively, compared with that under S0 treatment. Excessive nitrogen rate (N3) had significant effect on root ROC under lower salinity (S0 and S1), whereas significantly decreased ROC after booting stage compared to that of N2 treatment.

### The Na^+^ and K^+^ content and K^+^/Na^+^ ratio in rice root

**FIGURE 3:**
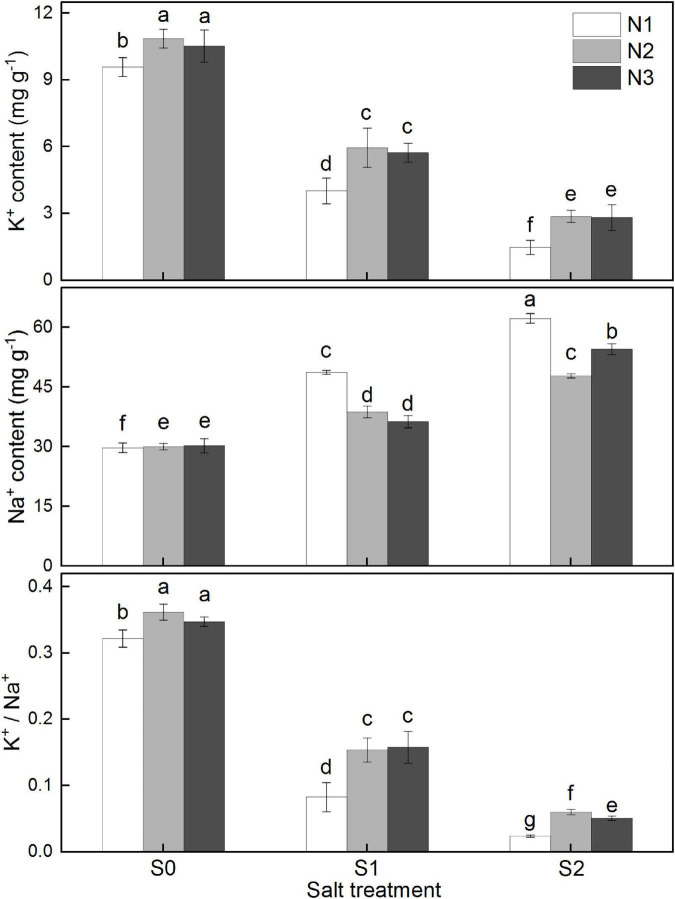
Effects of different salt concentrations and nitrogen rates on content of K^+^, Na^+^, and K^+^/Na^+^ ratio in rice root at full-ripening stage. S_0_, S_1_, and S_2_ denote salt concentrations of 0, 1.5, and 3.0 g kg^–1^ NaCl, respectively; N1, N2, and N3 denote nitrogen rates of 210, 300, and 390 kg ha^–1^. The data (*n* = 3) are mean values ± SD, calculated from three independent experiments. The different letters indicate significant differences at *P* < 0.05.

As shown in Figure 3, root Na^+^ content was significantly enhanced with the increase of salinity concentration, while K^+^ content and K^+^/Na^+^ ratio were significantly increased. Increasing the amount of nitrogen fertilizer significantly promoted the uptake of K^+^ by roots and inhibit the accumulation of Na^+^. However, excessive nitrogen fertilizer was not conducive to the K^+^/Na^+^ homeostasis in roots, mainly due to the enhanced uptake of Na^+^ under higher salinity (S2), which was significantly increased by 16.2% for N3, compared to that of N2 treatment.

### Root antioxidant enzyme activities

**FIGURE 4:**
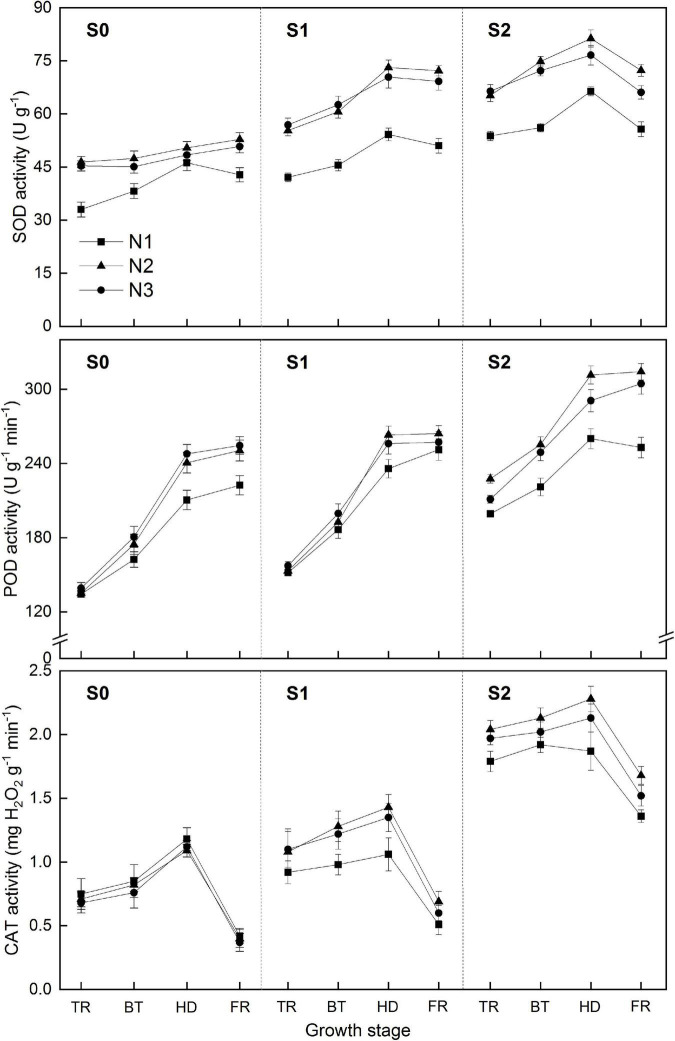
Effects of different salt concentrations and nitrogen rates on activities of SOD, POD, and CAT in rice root. S_0_, S_1_, and S_2_ denote salt concentrations of 0, 1.5, and 3.0 g kg^–1^ NaCl, respectively; N1, N2, and N3 denote nitrogen rates of 210, 300, and 390 kg ha^–1^. The data (*n* = 3) are mean values ± SD, calculated from three independent experiments. The different letters indicate significant differences at *P* < 0.05.

The root SOD activity was notably enhanced under salinity, while it was not well maintained under lower nitrogen rate (Figure 4). The average activity of SOD across the four growth stages was increased by 30.4 and 47.6%, respectively, for S1 and S2 treatment. Higher nitrogen rate was conductive to the maintenance of SOD activity. The average activity of SOD across the four growth stages was increased by 28.5 and 24.8%, respectively, for N2 and N3 treatment, compared to that of N1 treatment. However, excessive nitrogen fertilizer was not benefit to SOD activity under higher salinity (S2), where a significant decrease (by 5.8%) at heading stage occurred between N2 and N3 treatment.

POD activity increased gradually with growth stage and was significantly increased under salt stress. Nitrogen rate and combined effects of nitrogen and salinity were consistent to those of SOD activity. Notably, the POD activity under higher salinity (S2) was also lowered for N2 treatment compared to that of N1 treatment, after heading stage.

The CAT activity declined gradually with growth stage, and similar to that of SOD and POD, was significantly enhanced under salt stress. Concomitantly, excessive nitrogen fertilizer was not benefit to CAT activity under higher salinity (S2), where a significant decrease (by 5.8%) after booting stage occurred between N2 and N3 treatment.

### Leaf net photosynthesis rate

**FIGURE 5:**
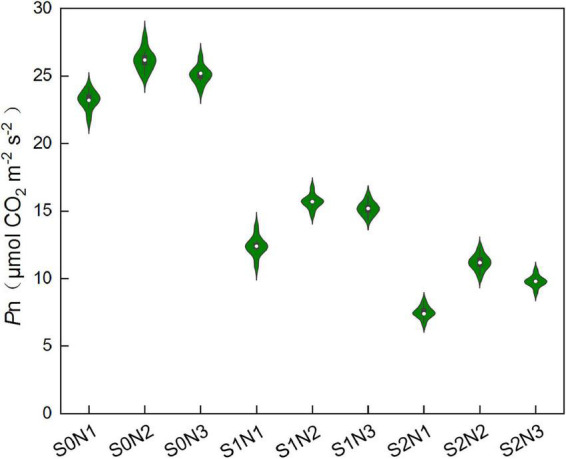
Effects of different salt concentrations and nitrogen rates on leaf net photosynthesis rate (*P*n) of rice at heading stage. S_0_, S_1_, and S_2_ denote salt concentrations of 0, 1.5, and 3.0 g kg^–1^ NaCl, respectively; N1, N2, and N3 denote nitrogen rates of 210, 300, and 390 kg ha^–1^. The data (*n* = 9) are mean values ± SD, calculated from nine independent experiments.

Salinity had significant adverse effect on leaf net photosynthesis rate (*P*n), while higher nitrogen was benefit to maintain relative high *P*n (Figure 5). The average *P*n was decreased by 41.9 and 61.7% under S1 and S2, respectively, compared with that under S0 treatment. Under lower salinity (S0 and S1), there was no notable difference between N2 and N3 on leaf *P*n, whereas N3 significantly lowered *P*n value compared to that of N2 under higher salinity (S2).

### N metabolism enzyme activity

**FIGURE 6:**
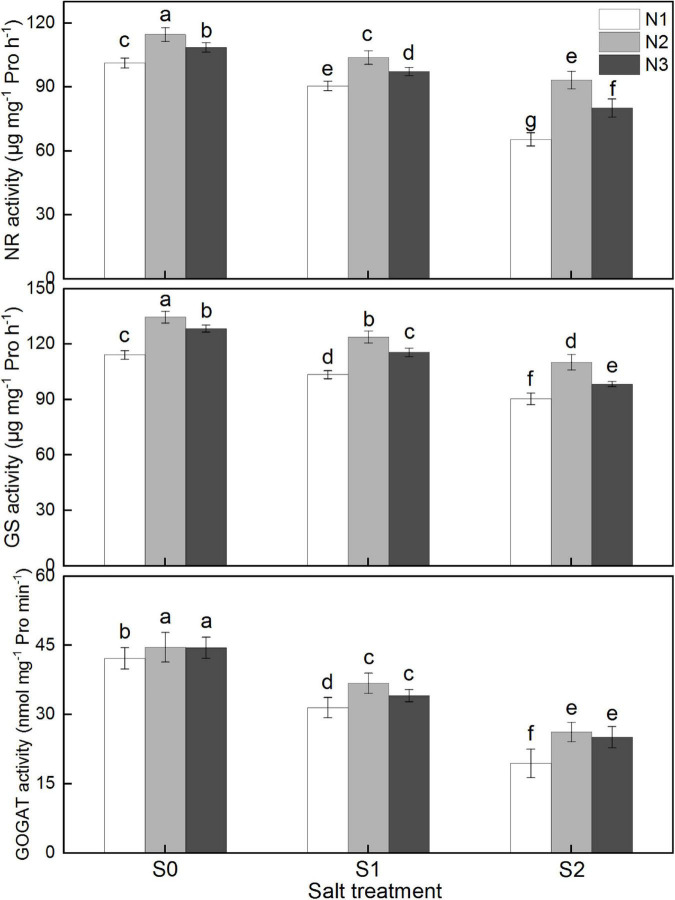
Effects of different salt concentrations and nitrogen rates on NR, GS, and GOGAT activities in rice leaf at heading stage. S_0_, S_1_, and S_2_ denote salt concentrations of 0, 1.5, and 3.0 g kg^–1^ NaCl, respectively; N1, N2, and N3 denote nitrogen rates of 210, 300, and 390 kg ha^–1^. The data (*n* = 3) are mean values ± SD, calculated from three independent experiments. The different letters indicate significant differences at *P* < 0.05.

As shown in Figure 6, salinity significantly decreased NR, GS, and GOGAT activities in rice leaf, while higher nitrogen was conductive to maintain relative high nitrogen metabolism activities. There was no significant difference on GOGAT activity between N2 and N3 treatment under varied salinity levels, whereas NR and GS activities were notably decreased (*P* < 0.05) for N3 treatment compared to those of N2 treatment. N3 treatment significantly decreased NR and GS activity by 5.3, 6.4, and 14.1%, and 4.6, 6.6, and 10.7%, respectively, under S0, S1, and S2 treatments, compared to N2 treatment.

## Discussion

### Effect of different nitrogen application rates on rice yield under salinity conditions

Nutrient management, especially nitrogen, is the most practical and easiest way of combating the salt stress. Rice plants experience nutrition efficiency due to poor uptake and transport of some essential nutrients like N, K, Ca, and Mg grown under salinity conditions leading to decreased yield or productivity (Hussain et al., 2012, 2013; Elkhoby et al., 2015; Zhang et al., 2022). Therefore the proper nutrient management occurs to be the most direct and realistic way of enhancing rice performance in saline area. As reported, rice plants exposed to salt stress decreased under N deficiency due to antagonistic effects of Cl^–^ with NO_3_^–^ ions (Ahanger et al., 2019). The grain yield was substantially decreased with increasing soil salinity; however, N application significantly counteracted the damaging effects of salinity on the paddy yield (Murtaza et al., 2000). It’s reported that rice grain and straw yield were linearly decreased under salinity, due to a notable decrease in number of tillers (Hussain et al., 2018). The higher salinity level of 16 dS m^–1^ observed a decrease of 50–52% and 38–48% in grain and straw yield, respectively (Singh et al., 2013). In the current study, a significant decrease of 19.7–51.7% was also observed under salinity stresses in average rice grain yield (Table 2). And inconsistent with previous study (Zhang et al., 2022), spikelets per panicle occurred to be the main factor affecting rice yield.

Researchers advocated that crops grown on sodic soils require 14–20% more N compared to that for normal soils (Oshaug, 2010), and numerous papers emphasized that the role of increased nitrogen supply in alleviating salinity toxicity for plant growth (Pramanik and Mandal, 2000; Irshad et al., 2008; Singh et al., 2008). Murtaza et al. (2000) reported that N application at the rate of 150 mg kg^–1^ of soil remained valuable to grow rice even at higher levels of soil salinity. In our field study, we found that final rice grain yield peaked at 300 kg ha^–1^ nitrogen application rate (Table 1). Interestingly, increasing nitrogen supply above 300 kg ha^–1^ failed to obtain a linearly increased rice yield. This was consistent to studies focusing on effects of nitrogen levels on rice yield in normal paddy field, while it was less concerned in saline field. Due to the huge difference between normal soil and saline soil, the mechanism of rice yield in response to different nitrogen levels should be quite different.

### Root physiological and morphological characteristics were essential for salinity tolerance

Plant roots possess uptake systems for both nutrients and water. Salinity adversely affected root development, while nitrogen was conductive to root morphology formation (Cheng et al., 2010; Chen et al., 2019). In our study, rice root dry weight, root length and root surface area were significantly decreased under NaCl treatment, and the magnitude of reduction increased with increasing salt concentration, whereas increased nitrogen rate significantly improved root morphological traits (Table 3). It’s reported that root biomass was positively correlated with crop yield (Munns and Tester, 2008; Annunziata et al., 2016); also, higher root length density, longer root total length and larger root surface area were vital in high yield formation (Dong et al., 2010; Chu et al., 2014). According to our results, changes of root morphological traits were consistent to changes of grain yield (Table 2) and leaf *P*n (Figure 5) in response to nitrogen under varied salinity levels, further indicating that superior root morphology was the agronomic basis for high crop productivity. Under saline conditions, insufficient nitrogen supply failed to afford the normal development of rice root and grain quality formation. However, excessive nitrogen was also not conductive, especially under higher salinity levels. Thus, there must be some critical physiological mechanisms determining the varied effects of nitrogen levels.

ROC is an important physiological indicator reflecting root vigor (Armstrong, 1967). Due to the close relation to the release of oxygen from roots, ROC can oxidize harmful reducing substances in the rhizosphere soil, thereby ensuring the normal metabolism of roots (Yamaguchi and Tanaka, 1990). Therefore, ROC of rice plays an important role in preventing excessive production of reductive toxic substances such as ferrous iron and hydrogen sulfide in paddy fields, promoting the absorption of nutrients and improving root metabolism, and protecting rice roots (Gutierrez et al., 2014; Onyango et al., 2020). Under saline conditions, rice ROC was significantly inhibited, while increased nitrogen rates improved the inhibition of salinity (Figure 2). However, ROC was not maintained high when nitrogen supply was excessive under higher salinity levels, indicating that coeffects of high nitrogen and high salinity may brought some internal physiological changes in root cells.

Salinity induces the risk of oxidative damage by stimulating production of ROS in plants cell. Overproduction of ROS might have occurred due to strong inhibition of photosynthetic activities. To cope up with oxidative damage, plants developed up-regulating strong enzymatic and non-enzymatic antioxidants (Parida and Das, 2005; Nath et al., 2016). Earlier it has been reported that enhanced activities of SOD, POD, and CAT functioning prevent the stress triggered oxidative damage (Nahar et al., 2016; Ahmad et al., 2018). SOD is indispensible for the dismutation of superoxide radicals in decreasing damages to photosynthetic machinery (Ahmad et al., 2010). Increased antioxidant activities help to reduce salinity mediated damage to membranes, proteins, nucleic acids and hence maintaining the functional and physiological stability (Ahanger et al., 2019). When nitrogen was insufficient, rice failed to maintain high antioxidant activities to cope with oxidative damage, while sufficient N mediated improved antioxidant potential resulted in alleviation of salinity induced oxidative damage to a significant level (Figure 4). However, excessive N was not conductive to maintain high antioxidants activities in high salinity condition (S2), which was inconsistent to that in normal paddy field.

It is well documented that disrupted K^+^/Na^+^ homeostasis occurs to be a major factor leading to growth inhibition and yield decline in salt-affected crop plants. Na^+^ toxicity is strongly related to the plant’s ability to sustain the acquisition and allocation of K^+^ (Tester and Davenport, 2003; Chen et al., 2007). Our results were consistent with the conclusion that the cytosolic K^+^/Na^+^ ratio is a key determinant of crop salinity tolerance (Figure 3). Increased nitrogen significantly decreased Na^+^ uptake and enhanced K^+^ absorbance in rice roots, thereby improving root K^+^/Na^+^ homeostasis. Our results are also in consonance with the finding of Ahanger et al. (2019) who reported that increased N supplementation resulted in significant increase in the uptake of N and K accompanied by reduced Na accumulation. However, previous studies have only suggested that increasing nitrogen supply can alleviate the effects of salt stress without clarifying the effect of high N under high salinity levels (Singh et al., 2019). In our study, excessive nitrogen did not show any significant impacts on K^+^ content even under higher salinity level, whereas Na^+^ was more accumulated by excessive N under relative high salt concentrations. Increased Na^+^ and Na^+^/K^+^ ratio may help to explain the effects of excessive nitrogen on root ROC and antioxidants activities.

### Effect of different nitrogen application rates on N metabolism under salinity conditions

Inorganic nitrogen is absorbed and transported by specific transfer proteins, such as ammonium transporters (AMTs) and nitrate transporters (NRTs) (Xu et al., 2012). For many plant species, only few nitrate is assimilated in roots, while a larger portion is assimilated in leaves (Black et al., 2002). In higher plants, NR is a rate-limiting enzyme in N assimilation that can directly regulate nitrate reduction, thereby regulating nitrogen metabolism (Masclaux-Daubresse et al., 2010). The ammonium, converted from nitrite by nitrite reductase is incorporated into amino acids, mainly through glutamine synthase (GS) and GOGAT (Kant et al., 2011). GS is reported a good candidate as a crucial and possibly rate-limiting enzyme in ammonium assimilation (Xu et al., 2012), and GS/GOGAT cycle is the main way to assimilate NH4^+^ in rice (Gu et al., 2020).

NR, GS, and GOGAT display sensitive to salinity and their decreased activities have been observed in plants under saline stress (Katiyar and Dubey, 2010; Rohilla and Yadav, 2019; Gu et al., 2020). It’s reported that the declines of activities of NR, GS, and GOGAT in rice leaves were greater than those in roots at heading stage under salt stress (Gu et al., 2020). Our results showed that activities of the three enzymes were all significantly declined under salinity, and the decline amplitude was greater with the increasing salinity levels. Declines N metabolism enzymes inevitably inhibited the regulation and uptake of N, which would further adversely affect leaf photosynthesis and grain yield formation (Ali et al., 2020).

Most papers addressed that increased N supply could effectively enhance N metabolism activities and increase nitrogen uptake and total content in plants, whereas excessive nitrogen was not conductive to NR and GS activities (Figure 6). Reduced N metabolism was one of the important reason explaining that high N application failed to obtain the ideal photosynthetic rate and final grain yield (Cisse et al., 2020).

## Conclusion

Saline land is an important reserve arable land resource for whole humans. Most Saline soils are infertile and lack various nutrients suitable for plant normal growth. In contrast, saline soils are rich in sodium ions, chloride ions, etc., which would inhibit plant roots absorbing nutrients and water from soils. Most of the previous papers elaborate that increased nitrogen supply can effectively make crops grow stronger to resistant to salinity stress. However, this study demonstrates that excessive nitrogen input is not conductive to increased grain yield, especially in higher salinity conditions. Our results showed that the appropriate nitrogen application rate was 300 kg ha^–1^ with the grain yield of 7.7 t ha^–1^ in saline soil (the conductivity was 5.5–6.2 mS cm^–1^). Insufficient nitrogen rate under saline conditions failed to maintain high root biomass, root length and surface area, ROC, K^+^/Na^+^ homeostasis, activities of antioxidant enzymes and nitrogen metabolism-related enzymes, which inevitably lead to decreased grain yield. In accordance with previous studies, we found increased nitrogen supply indeed enhanced leaf photosynthesis and grain yield of rice under salt stress, mainly due to the improved root morphological and physiological characteristics. Under lower salinity, the notably decreased rice grain yield with excessive N application was mainly attributed to decreased activities of NR and GS. However, the disrupted K^+^/Na^+^ homeostasis due to increased Na^+^, decreased antioxidant enzyme activities and NR and GS activities worked together to decreased leaf *P*n and rice yield resulted from excessive N under higher salinity level. Overall, our results suggest that superior root physiological and morphological characteristics play vital role in determining rice salinity tolerance. Thus, to further clarified the detailed pathway, the molecular mechanisms of nitrogen application rate on rice production in saline soil will be recommended in the future work.

## Data availability statement

The original contributions presented in this study are included in the article/supplementary material, further inquiries can be directed to the corresponding author.

## Author contributions

YC and QD planned and designed the experiments. YC, YL, RZ, YZ, and RL performed and recorded data during the experiments. YC, JG, and HW statistically analyzed the data and prepared the tables and graphs. YC wrote the manuscript. YC, ZH, KX, and QD approved the final manuscript after review. All authors have read and agreed to the published version of the manuscript.
